# P-2033. Documentation of Candidemia Risk Factors for T2 Ordering: A Diagnostic Stewardship Intervention

**DOI:** 10.1093/ofid/ofaf695.2197

**Published:** 2026-01-11

**Authors:** Kenneth D Long, Todd P McCarty, Peter G Pappas, Sixto M Leal, Joshua Stripling

**Affiliations:** The University of Alabama at Birmingham, Birmingham, Alabama; University of Alabama at Birmingham, Birmingham, AL; University of Alabama at Birmingham, Birmingham, AL; University of Alabama at Birmingham, Birmingham, AL; University of Alabama at Birmingham, Birmingham, AL

## Abstract

**Background:**

Candidemia is a highly morbid condition that prompt initiation of antifungal therapy can decrease by as much as 50%; however, there is low incidence in patients without specific underlying risk factors. The T2Candida diagnostic measures Candida-specific DNA in whole blood, providing results in ∼4 hours, but cannot differentiate between viable (living) Candida and circulating DNA, which can persist for 14 days. We assess the utility of a clinical decision support system (CDSS) comprised of a popup requiring selection of risk factors before allowing a test to be ordered.

**Figure:**
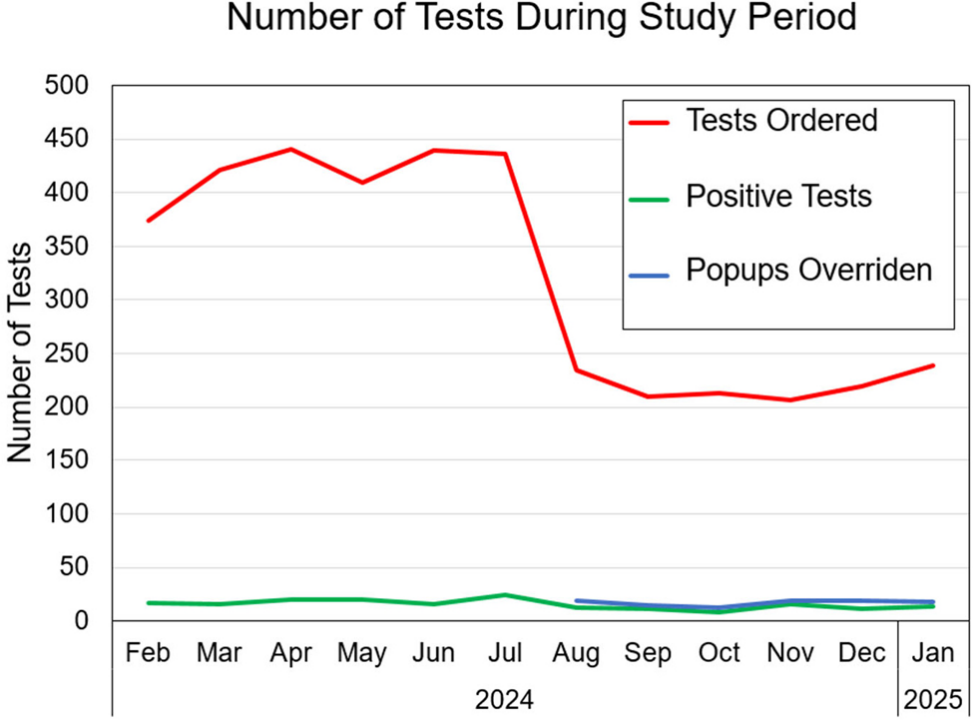

**Figure:**
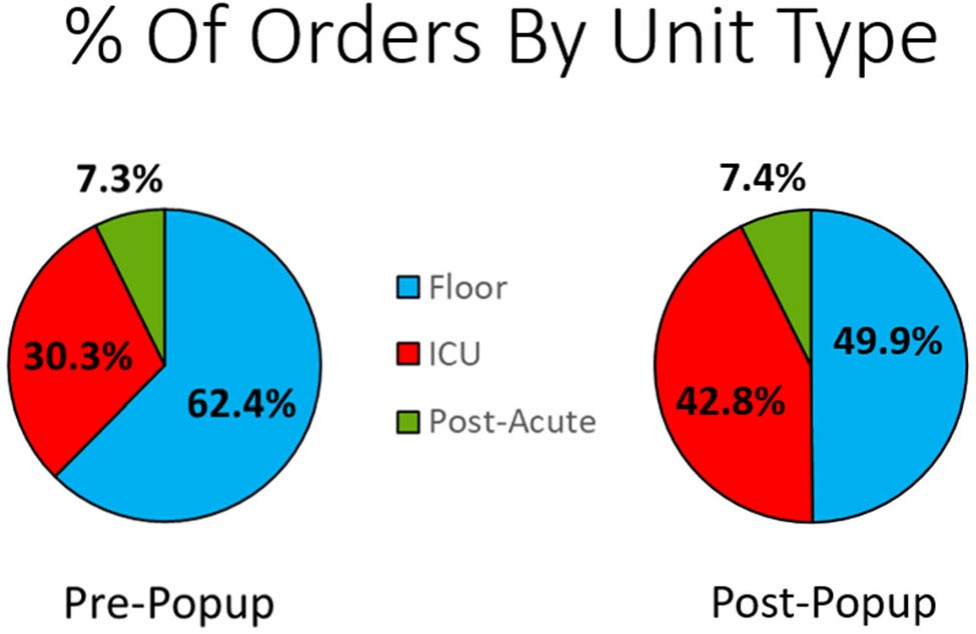

**Methods:**

A prior informational CDSS was modified to require identification of multiple risk factors for Candidemia, otherwise it canceled the order. A deliberate override was included, but made to be challenging enough to dissuade most users. A retrospective analysis of all inpatient T2 tests ±6 months from CDSS implementation was conducted (n=3,842). Blood culture positivity and micafungin usage were analyzed near T2 order. Blood culture positivity was used as a diagnostic gold-standard at ±12h (false negative) and ±7d (false positive).Univariate and multivariate analyses were performed.

**Figure:**
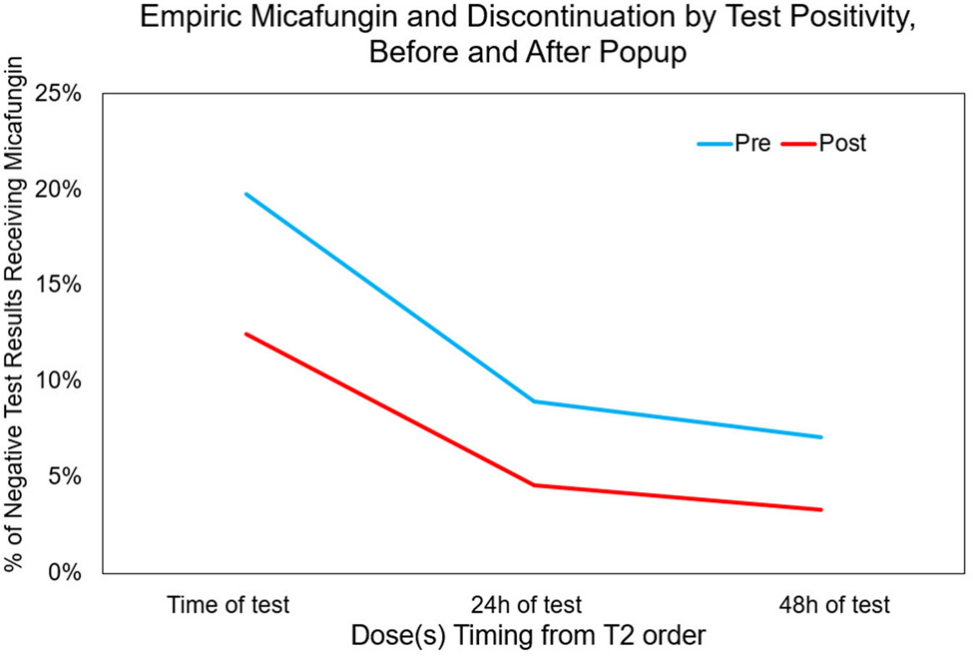

**Figure:**
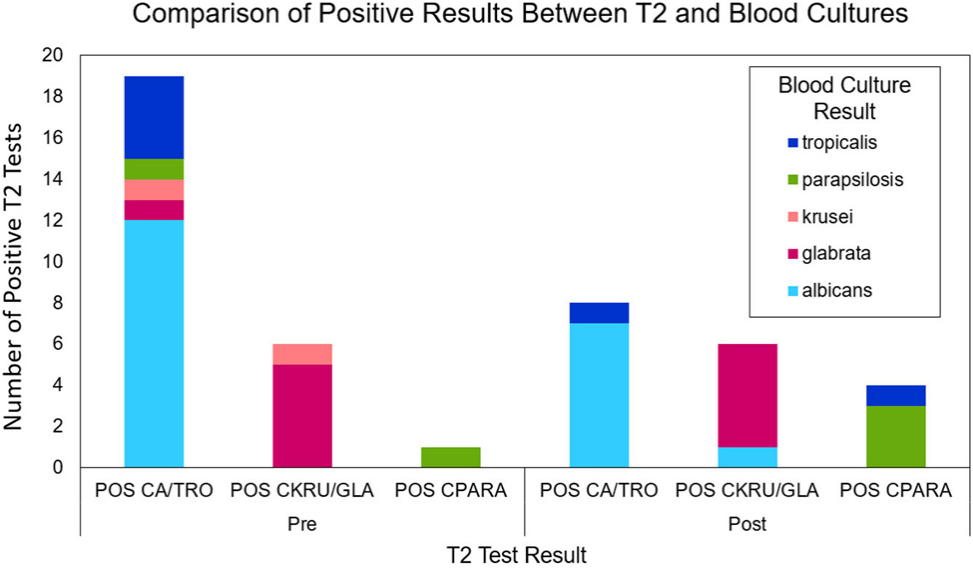

**Results:**

The new CDSS launched in August 2024, resulting in a rapid 47.5% reduction in T2 orders, equivalent to $540k annual savings (Fig. 1). The overall test positivity rate was increased, but not significantly (5.6% vs. 4.5%). The distribution of tests ordered among inpatient unit types (ICU vs. Floor vs. Post-Acute) changed significantly with the highest reduction in testing from Floor units (Fig. 2, p< .0001).103 popups were overridden, yielding 88 completed tests. 5 of these were positive; 100% of which had ≥ 2 sets of negative blood cultures drawn ±12h. 15-20% of patients received empiric micafungin at time of T2 with a higher proportion of appropriately discontinued therapy in the post-CDSS cohort (Fig. 3, p =.018). Differences in identified species between positive T2 and blood cultures are shown in Fig. 4.

**Conclusion:**

A CDSS that requires correct identification of underlying risk factors decreased the number of ordered tests by almost 50%. A significant decrease in tests ordered for patients on the floor compared to those in an ICU (a risk factor addressed in the CDSS) was observed, indicating test reduction in a lower risk population.

**Disclosures:**

Todd P. McCarty, MD, Basilea: Grant/Research Support|Cidara: Grant/Research Support|F2G: Grant/Research Support|Mundipharma: Grant/Research Support|Pfizer: Advisor/Consultant|Scynexis: Grant/Research Support Peter G. Pappas, MD, Astellas: Grant/Research Support|Basilea: Advisor/Consultant|Basilea: Grant/Research Support|F2G: Advisor/Consultant|Gilead: Grant/Research Support|Melinta: Advisor/Consultant

